# American Medical Association

**Published:** 1853-05

**Authors:** 


*American Medical Association.*

FROM the *New York Daily Times* we are able to make up an abstract of the proceedings of this body at their sixth annual meeting.

The American Medical Association held their sixth annual meeting, May 3, in the Presbyterian Church, Bleeker-street, the President, Dr. Beverly Wellford, in the Chair. The morning was occupied by the Committee of Arrangements, in receiving delegates from the several States. At 11½ A.M., the meeting organized, and the President welcomed the Delegates to the City. There were nearly five hundred gentlemen present.

After a congratulatory address by Dr. F. Campbell Stewart, chairman of committee of arrangements, Dr. Pope, of Missouri, extended an invitation to the Association to hold their next unnual meeting, for 1854, at St. Louis.

Dr. Conde, of Pennsylvania, said he was instructed to invite the Association to hold their next annual meeting in Philadelphia.

Dr. Hays, of Pennsylvania, although desiring that the Association would meet in Philadelphia, was forced to give his voice for next year in favor of St. Louis. He hoped that at their next meeting, the claims of Philadelphia would be remembered.

The motion to meet at St. Louis was put and carried.

Dr. Conde, of Pennsylvania, Chairman of Committee on Publications, submitted a Report from the Committee, with resolutions appended, making the assessment for the present year $5 ; authorizing the Committee to decide upon the terms at which the volume of Transactions for this year shall be furnished; and further authorizing them to take such measures in relation to disposal of the copies as they may deem expedient. Dr. Conde stated that a valuable paper would be submitted at this meeting, the mere illustrations of which would cost $1,000 for printing.

The resolutions were adopted.

The Treasurer's Report showed the total receipts for the past year to be $1,905; paid out $2,015; balance due to Treasurer, $100.

On motion of Dr. Conde, the Committee was authorized to furnish the Chairman of Committees on Epidemics with extra copies of their reports, respectively, at the expense of the Association.

The Secretary read communications inviting the Association to visit University Medical College, the Anatomical Museum, and Bloomingdale Lunatic Asylum.

Dr. F. C. Stewart presented a report, recommending the admission of Dr. Marshal Hall, of London, Surgeon Mower, U. S. A., Surgeons Bache, Pinckney, Brownell and Simyson, U. S. N., Drs. Leonard and Betton, Florida, Hon. Dr. Bartlett, N. Y. Senate, Dr. Harris, Canada, Dr. Rodder, Canada West, Drs. McIlvaine and Pittman, American Medical Society, Paris, to participate in the proceedings by invitation.

On motion of Dr. Cox, a Committee was appointed to wait on Dr. Marshal Hall, and conduct him to a seat on the platform.

The President then read a lengthy and very able address, reviewing the origin, progress and benefits achieved by the Association.

Dr. Hays, of Pennsylvania, moved the thanks of the meeting be presented to the President for his elegant, appropriate and eloquent address, and requesting a copy for publication in the Transactions of the Association. Carried.

The Secretary read a resolution passed by the Medical Society of Virginia, recommending the appointment of a well-qualified chemist to analyze the most most prominent nostrums of the day, and publish the results monthly in the leading newspapers of each Sta te. Also, a communication from the President of the American Medical Society at Paris, appointing Drs. Pittman, Walton and McIlvaine to attend this meeting.

On motion of Dr. Atley, the Committee on Publications were directed to send a full set of Transactions of the Association to the Society in Paris.

A communication was received from Dr. Ramsay, of Georgia, enclosing documents on personal matter, which were laid on the table.

The Committee on nominations reported the following officers for the ensuing year:

*For President*—Dr. Jonathan Knight of Conn.

*Vice-Presidents*—Drs. Usher Parsons, of R. I., Lewis Condict, of N. J.; Henry R. Frost, of S. C.; R. L. Howard of Ohio.

*Secretaries*—Drs. Edward L. Beadle, of N. Y.; and Edwin L. Lemonie, of Missouri.

*Treasurer*—Dr. Francis Conde, of Penn.

The Committee reported St. Louis, Mo., as the place to hold the next annual meeting.

The report was adopted.

Drs. Gooch, Watson and Atlee were appointed a Committee to conduct the President elect and other officers to their seats.

Dr. Knight, on taking the Chair, returned thanks for the honor conferred on him.

Dr. Atlee, of Pennsylvania moved a vote of thanks to the late President, Dr. Wellford, for his dignified, courteous and efficient manner in the Chair. Carried unanimously, the members rising.

A vote of thanks was also passed to the retiring Secretary, Dr. Gooch.

On motion of Dr. Hopkins, of Maryland, it was resolved that no member speak more than ten minutes on any subject at one time.

Dr. Stewart, on part of Committee of Arrangements, suggested that the Association meet each day at from 9 to 12 A. M., and from 1 to 4 P. M.

On motion of Dr. Gooch, the meeting adjourned to 9 A. M., to-day (Wednesday.)

*May* 4.—The Association met in Bleecker street Presbyterian Church, at 9. A. M. There was a large attendance of delegates.

Dr. E. L. Beadle, Secretary, read the minutes, which were adopted.

Dr. Cox, of Maryland, moved for a reconsideration of the vote adopting the minutes, to allow of a correction in the Special Report of Committee of Arrangements, inviting Drs. Pickney and Bache, U. S. N., to take part in the proceedings. These gentlemen had a right to sit as delegates from the Army and Navy.

Dr. Steward, explained, and read from the 2d. article of the Constitution relating to delegates.

Dr. Watson, of New York, stated they had been always received as delegates.

The motion to reconsider was put and lost.

Dr. Cox, hoped the Association would not take any action that would give offence to the Army and Navy Medical Institutions.—He moved that Drs. Pickney and Bache be received as regular delegates.

Dr. Pickney, U. S. N., asked to be heard and claimed his right to a place as delegate, having been formerly received as such and signed the Constitution.

Dr. F. C. Stewart, offered the following as an amendment to Dr. Cox's motion.

*Resolved*, As the sense of this Association, under the present Constitution delegates can be received from the U. S. Army and Navy Medical Bureaux when appointed by the Chief of the Army and Navy Medical Bureaux.

The amendment was adopted.

Dr. C. D. Meigs, of Philadelphia, presented a report on "Acute and Chronic Diseases of the Neck of the Uterus," with a request that it should be referred to Committee on publication without reading.

Dr. Condie of Pennsylvania, Chairman of Committee on Causes of Tubercular Diseases, stated that in consequence of his duties as Treasurer, and Chairman of Committee on Publication, he had never been able to complete his report. On motion of Dr. Atlee, the Committee was continued.

On the part of Dr. Porcher, of South Carolina, Dr. Condie stated that he had sent to him the report of Committee on "Toxicological and Medical Properties of our Cryptogamic Plants," but with the request that it be left for further additions by the Committee. The Committee was continued.

Dr. G. Emerson, of Pennsylvania, Chairman of Committee on "Agency of the refrigeration produced through radiation of heat, as an exciting cause of Disease," presented his report, which was referred to Committee on Publication, and an abstract read. The sanitary lesson designed to be inculcated in this paper is the importance of guarding against exposure to the refringerating effects of nocturnal radiation, especially in sickly places and during epidemic periods. The means of effecting this are shown to be extremely simple and always at hand, as any thing will answer the purpose which may be interposed to cut off the view of the open sky, and thus prevent "upward radiation."

Dr. H. F. Campbell, of Georgia, presented a report on Typhoid Fever, which was referred to Committee on Publication.

Dr. Sutton, of Kentucky, presented a report on epidemics of Tennessee and Kentucky, which was referred to Committee on Publication.

Dr. Pitchar, of Michigan, presented a report on the subject of Medical Education, which he was requested to read at length. The report was a long and able document, containing many valuable suggestions to prevent the spread of quackery, and on the best means of training the medical student. The committee proposed that all candidates for degrees shall have studied at least three years, and recommended the extension of the lecture seasons to six months. The committee repeated their high opinion of the benefits to be derived by students from bed-side experience, as superior to lectures and flitting hospital visits, and suggested a supplementary school of practice. They would not discourage medical schools through the country, but foster them as useful, and trust to the private instructor and the hospitals as schools of practice. The committee asked leave to conclude their report by presenting the following resolutions:

*Resolved*, That the Association re-affirm its formerly expressed opinions, on the value and importance of general education to the student and practitioner of medicine, and that it would gladly enlarge its rule on this subject, so as to include the humanities of the schools, and the natural sciences.

*Resolved*, That in the opinion of this Association, a familiar knowledge with the elements of medical science should precede clinical instruction.

*Resolved*, That in order to accomplish the latter, the hospitals, when elevated to the rank of schools of practice, and the intelligent private preceptor, are the most efficient instrumentalities to be used for that purpose.

On motion of Dr. Atlee, the report and resolutions were adopted.

Dr. J. M. Smith, of New York, chairman of Committee on Volunteer Communications, reported the receipt of two prize essays; the first, entitled, "The Cell—its Physiology, Pathology, and Philosophy," by Waldo J. Burnett, M. D., Boston, Mass.; the second, on the "Surgical Treatment of certain Fibrous Tumors of the Uterus, heretofore considered beyond the Resources of Art," by Washington L. Atlee, M. D., Philadelphia; and other papers.

Dr. Aldan March, of New York, made a verbal abstract of his paper on "Diseases of the Hip Joint," which was favorably reported on by the committee; and, on motion of Dr. A. Smith, he was requested to read the paper, during recess to-day, in Crosby-street Medical College.

Prof. Palmer, Chicago, moved the following:

*Resolved*, That this Association earnestly recommend to the local societies in different portions of our country, to appoint committees, whose duties it shall be to record the prevalence of epidemics or other diseases, and the general state of health in their respective localities, and to transmit said reports to the Committees of the Society on Epidemics, through the State Societies where they exist.

*Resolved*, That the Secretaries be requested to secure a wide publicity to the above resolutions, by such means as they may deem proper.

The resolutions were adopted.

Dr. Stewart read the following resolutions, offered by Dr. Stephen W. Williams. of, Mass., a permanent member:

*Resolved*, That the thanks of this Association be presented to Dr. Winslow Lewis, of Boston, a member of the Massachusetts Legislature, for the bill which he has presented and endeavored to sustain, providing that "no druggist, apothecary, or person engaged in manufacturing medicines, or compounds to be administered as medicines, (except such as are published in standard works of chemistry, materia medica, or pharmacopœia,) shall offer the same for sale in any way, till he has filed a complete recipe in English, sworn to before a legal authority constituted for such purpose."

2. *Voted*, That a committee be appointed by this Association for the purpose of petitioning Congress and State Legislatures to enact regulations and laws similar to the above.

Also, as we are constantly called upon to deplore the ravages of death among the illustrious and worthy members of our profession throughout the United States.

*Resolved*, That a Standing Committee be appointed by this Association, to procure memorials of the eminent and worthy dead among the distinguished physicians of our country, both in and without the pale of the Association, and present them to this Association for publication in their transactions.

The first resolution, relating to Dr. Lewis, was adopted.

To the second. Dr. Cox, Mo., moved an amendment, confining the memoirs to distinguished members of the Association.

Dr. Morgan, Washington, thought the Association would not be able to meet the increased expense such an addition to their transactions would cause. He moved to lay on the table. Carried.

Dr. Buck, N. Y., read a paper on morbid growths within the larynx, and exhibited a specimen, with report of the case. Referred to Committee on Publications.

Dr. Mitchell, Pa., offered the following:

*Whereas*, The claim of Naval Medical officers to a defined rank, assimilated with the grades of officers of the line of the navy, has not yet been decided by Congress, therefore,

*Resolved*, That the President of this meeting appoint a Committee of three, which is hereby instructed to communicate to Congress, through the presiding officer of each house, at the commencement of its next session, an expression of the interest felt by the American Medical Association of the United States, for their professional brethren employed in the navy, as set forth in resolutions unanimously adopted at several previous sessions of this body.

Dr. Jackson, of Philadelphia, seconded the resolution.

The President stated that the bill had passed one branch of our National Legislature.

Dr. Pinckney, U. S. N., made a statement of the action had in the National Legislature.

The resolution was put and adopted.

Dr. Stevens, of New York, moved that the President be Chairman of the Committee.

Dr. Hooker, of Connecticut, offered the following, which was adopted.

*Resolved*, That the delegates from the several States, be requested to appoint a Committee, who shall aid the Committee of Publication in procuring subscriptions and in distributing the annual transactions of the Association.

Dr. Bolton, of Virginia, moved a resloution expressing the warm commendation of the Association towards the medical department of Michigan University, for its co-operation in elevating the standard of the profession.

A discussion ensued in which Drs. Palmer and Bolton contended for the resolution, and Dr. Hooker Conecticut, and others, against it. Finally, the resolution was withdrawn.

A report was received from Dr. Simons. of South Carolina, recommending a memorial to Congress to appoint a surgeon on each emigrant vessel. The Committee report that a memorial was placed in charge of Dr. Jones, M. C., (and member of the Association,) of Onondaga Co., N. Y., to be presented to the House of Representatives. The report states that the memorial was quietly deposited in the archives of the Committee on Commerce, and received no notice from them whatever. The Committee was too busy to attend to such small matters as the lives of emigrants, while the weightier matters of President-making rested on their shoulders. Now that these important subjects are disposed of and the spoils mostly shared, there was hope that the memorialists might be treated with rather more consideration, and at their request the Committee was continued.

On motion of Dr. Atlee, the report was adopted, and the Committee continued·

Dr. Simmons also reported on a resolution by Dr. Sutton, requesting Congress to separate the medical census, for the convenience of the profession. Their petition was not granted, the gravest objection being the alledged inaccuracy of the medical statistics of the Census. The Committee urged renewed effort on the part of the Association to induce each of the State Legislatures to establish a Registration of Births, Deaths and Marriages, pointing for an illustration alike of what has been and what ought to be done to the Reports from Massachusetts.

The report was adopted, and the Commttee continued.

*May 5th.*—The Association met at 9 A. M., Dr. Jonathan Knight, President, in the Chair.

After the reading of the minutes, Dr. Hooker, Connecticut, offered the following, which was adopted:

*Resolved*, That a Committee of Five be appointed, whose duty it shall be, in compliance with the suggestions of our late President, Dr. Wellford, to report our plans of organization for State and County Societies, and that the Committee be requested to report, if possible, during the present meeting of the Association.

Dr. Zeigler, Pennsylvania, offered the following, which was referred to Committee on organizing State and County Societies.

Inasmuch as the universal aggregation and organization of the members of the medical profession in the United States has long been a desideratum, but partially attainable until the adoption of the present mode of organizing local and general societies in imitation of, and in conformity with, the confederacy of this country; and whereas, notwithstanding this consolidation has thus been more completely and perfectly effected, a very large portion of the profession still remain isolated and unassociated, thus retarding and preventing the more rapid attainment of those exalted objects involved in this universal professional union in one great brotherhood; and inasmuch as the present general mode of perfecting further and final professional aggregation and consolidation is comparatively imperfect, inefficient, and protracting, and hence will require an almost indefinite period for its full accomplishment, and final consummation and completion; and inasmuch as some more general and systematic effort for the speedy and positive realization of this highly important object is yet greatly desirable; therefore,

*Resolved*, That the American Medical Association hereby reiterate the repeatedly previously expressed desire for the immediate formation and organization of County and State Medical Societies in every part of the country in which they have not yet been established.

*Resolved*, That every County Medical Society be and is hereby recommended to dispense with the present system of acquiring members by the previous pro-formal manifestation of personal desire on the part of applicants for such association, and to substitute therefor that of the immediate and voluntary election to membership therein, of every unassociated eligible physician.

*Resolved*, That all physicians thus voluntarily elected, and subsequently neglecting or declining to respond to and unite themselves with the general profession, shall be considered as estimating their own personal views, and private relations and interests, above and in opposition to those of the profession generally, and, as thus antagonistic to its exalted objects, cannot therefore consistently expect the continued enjoyment of the usual rights and privileges of professional intercourse and fellowship.

The Secretary read a notice, requesting delegates from any

States which were not represented on the Committee of Nomination, to elect representatives, to sit with the Committee at 56 Bleecker-street.

Dr. N. S. Davis, of Illinois, reported at length, and lucidly, on the Medical Literature of 1853. There are now published in the United States twenty-eight medical periodicals, of which four are issued quarterly, six bi-monthly, fifteen monthly, two semi-monthly, and one weekly. One of the monthlies is published in the German, at New York, and one in French, at New Orleans. Of the aggregate number of pages published, about one-half were original matter. This aggregate consists of the record of cases occurring under the observation of their writers, of which a very large proportion lose their value for lack of that fullness of detail and scope which are essential to make them reliable data for the abstraction of practical deductions—articles embodying the statistical results of certain diseases and surgical operations, and essays on special subjects, and the details of experimental inquiries—of all which classes, some of the more important specimens were named in the report.

Many other interesting subjects were embraced in the report, for which we have no space in the present number.

Dr. Yandell, of Kentucky, offered the following, which was adopted:

*Whereas*, By the dispensation of an inscrutable Providence, Dr. Daniel Drake has been removed since the last meeting of this Association, from the scene of his earthly labor.

*Resolved*, That, in the death of Dr. Drake, the American Medical Association has lost one of its most honored members, and the American Medical profession one of its brightest ornaments.

*Resolved*, That his steady devotion to his profession through a long life, his zeal, activity, and unceasing efforts to advance its interests, afford an example worthy of the imitation of every young physician.

*Resolved*, That this Association will cherish the memory of Dr. Drake for his many virtues, and for his labors which have adorned and elevated our profession.

The resolutions were adopted by a rising vote.

Dr. Condie, of Pennsylvania, and Dr. Cox, of Maryland, offered the following resolutions, which were adopted by a rising vote:

*Resolved*, That we have heard with sincere regret of the death of our late fellow member, Dr. Isaac Parrish, of Philadelphia, who was distinguished by his early and earnest advocacy of the establishment of this Association, by his ardent interest in its proceedings, and by his valuable contributions to its published proceedings.

*Resolved*, That in the demise of Dr. William E. Homer, which has occurred since the last annual session of this body, the American Medical Association has lost one of its illustrious and useful members; and the science of medicine, an indefatigable student and most distinguished teacher.

*Resolved*, That the memory of the gifted subjects of the resolutions, dear as it must ever be to the lovers of medical science universally, will be especially cherished by this Association, to whose great objects and aims, their last efforts were, during life, promptly and liberally bestowed.

Dr. Condie, of Pennsylvania, replied to inquiries received, in reference to back numbers of the transactions.

Dr. Yandell, of Kentucky, presented a report received from Dr. S. D. Gross, Kentucky, on the results of surgical operations, for the relief of malignant diseases, which was referred to a Comm ttee on publication.

Several resolutions of interest were discussed. The proposed amendments to the constitution, with the exception of the clause relating to delegates from the army and navy, were indefinitely postponed.

The Committees will be given in our next. J.

